# Forensic Support for Abraham et al.’s BB Protocol

**DOI:** 10.3390/e27050504

**Published:** 2025-05-08

**Authors:** Qidi You, Hongjian Yang, Xiyong Zhang, Xiaotong Jiang, Kaiwen Guo, Kexin Hu

**Affiliations:** 1 Beijing Institute of Satellite Information Engineering, Beijing 100086, China; youqd@spacestar.com.cn (Q.Y.); xiyong.zhang@hotmail.com (X.Z.); 19946284097@163.com (X.J.); 2 Institute of Software, Chinese Academy of Sciences, Beijing 100190, China; kaiwen2016@iscas.ac.cn (K.G.); hukexin@iscas.ac.cn (K.H.); 3 University of Chinese Academy of Sciences, Beijing 100049, China; 4 Zhongguancun Laboratory, Beijing 100086, China

**Keywords:** blockchain, distributed systems, consensus, fault tolerance threshold, Byzantine nodes, forensic support

## Abstract

The consensus protocol is a fundamental building block in distributed computing and has been widely used in blockchain systems in recent years. Paxos, introduced by Lamport, stands out as one of the most widely adopted consensus protocols and has found application in renowned distributed systems, including Google’s Spanner system. Abraham et al. analyzed the FaB Paxos protocol, a Byzantine version of Paxos. They abstracted the single-shot FaB Paxos into a Byzantine broadcast protocol and further gave an enhanced protocol known as Abraham et al.’s BB. Abraham et al.’s BB protocol achieved optimal two-round message interaction under good conditions, satisfying the optimal fault tolerance threshold of n=5t−1 where *n* represents total number of nodes in the system and *t* denotes the tolerable number of Byzantine nodes. This paper delves into scenarios wherein the actual number of Byzantine nodes surpasses the fault tolerance threshold during the operation of Abraham et al.’s BB protocol. To address this, we propose a forensic protocol designed to offer forensic support in cases of agreement violations. The forensic protocol aims to label Byzantine nodes through irrefutable evidence. We analyze the forensic protocol, elucidating the number of Byzantine nodes that the forensic protocol can label under different circumstances, along with the corresponding number of required messages. Additionally, we present an impossibility result, indicating that forensic support for Abraham et al.’s BB is impossible when the number of Byzantine nodes exceeds 2t−2.

## 1. Introduction

The Byzantine Fault-Tolerant Broadcast Protocol [1] enables multiple mutually untrusted nodes to reach consensus on the broadcast value of a particular message sender, making it a vital research area in distributed systems. Its applications are extensive, particularly in secure multi-party computation [2] and blockchain systems [3].

The Byzantine Fault-Tolerant Broadcast Protocol is often used to construct Byzantine State Machine Replication Protocols, which are core components of blockchain systems. Most existing protocols follow a master-backup design, where the protocol execution is divided into views. Each view has a designated leader responsible for issuing a broadcast value and guiding other nodes to consensus. Thus, each view’s protocol flow can be regarded as a Byzantine broadcast protocol, with the view leader acting as the broadcaster. Researchers can leverage efficient Byzantine fault-tolerant broadcast protocols to build efficient Byzantine state machine replication protocols [4].

Performance is critical in blockchain systems. Recent research [5,6,7] has focused on reducing the number of interaction rounds to lower latency. Dolev et al.’s work [8] shows that, in the worst case, Byzantine fault-tolerant broadcast protocols require at least t+1 rounds, where *t* is the number of Byzantine nodes. Here, *t* (the fault tolerance threshold) is generally proportional to the total number of nodes *n*. However, in practice, t+1 rounds can cause excessive latency, making it unsuitable for real-world applications. Consequently, research increasingly focuses on optimizing protocols for “good cases” wherein the leader is honest and the network is synchronous.

The PBFT protocol [5], designed by Liskov et al., was the first practical Byzantine state machine replication protocol. Under good conditions, it achieves consensus in just three rounds of message interactions. Later, Martin et al. introduced the FaB protocol [6], which further reduces the interaction rounds to two under good conditions. However, this improvement comes at a cost: the fault-tolerance threshold decreases from n=3t+1 to n=5t+1.

At PODC 2021, Abraham et al. improved upon FaB by abstracting the single-consensus FaB protocol into a Byzantine broadcast protocol and presenting an enhanced protocol, Abraham et al.’s BB [4]. Abraham et al.’s BB protocol, while achieving the good case of two rounds of message interactions, raises the fault-tolerance threshold to n=5t−1. That is, to tolerate *t* Byzantine nodes, the total number of nodes *n* in the protocol must satisfy n≥5t−1. Furthermore, Abraham et al. proved that n=5t−1 is the optimal fault tolerance threshold for a semi-synchronous Byzantine broadcast protocol with two rounds of message exchanges. Although Abraham et al.’s BB achieves the optimal fault tolerance threshold for two-round interactions, its threshold is lower than that of classic Byzantine protocols with three-round interactions, such as PBFT [5], which have a threshold of n=3t+1. If the actual number of Byzantine nodes exceeds the protocol’s fault tolerance threshold, the protocol cannot guarantee security. Therefore, the lower fault tolerance threshold of Abraham et al.’s BB presents a potential security risk, highlighting the need for a solution to address this issue.

Providing forensic support [9] for Abraham et al.’s BB protocol is one solution. Forensic support focuses on the number of nodes controlled by the adversary that exceed the fault tolerance threshold of the protocol, thus causing a violation of the protocol’s agreement. Specifically, the forensic protocol is designed to identify Byzantine nodes engaging in malicious behavior when the protocol violates agreement. With the forensic protocol, the system can quickly detect problematic nodes and take appropriate actions, such as removing or restarting the labeled nodes. This helps prevent security issues from recurring in subsequent rounds of consensus. Additionally, in a proof-of-stake-based blockchain system, forensic support allows the system to hold malicious nodes accountable by reducing their stakes or margins. Similar recourse mechanisms are used in several blockchain protocols, such as the Gasper protocol [10] adopted by Ethereum and the Tendermint [11] consensus protocol in the Cosmos system, both of which provide recourse for violating nodes. It is important to note that this paper does not alter the consensus process of Abraham et al.’s BB protocol; it preserves its original structure. The forensic process only requires nodes to record specific messages during protocol operation as evidence to identify Byzantine nodes.

The comparison between the protocol designed in this paper and the forensic protocols designed by Sheng et al. [9] for two Hotstuff variants (HotStuff-view and HotStuff-hash) is presented below.

Compared to the forensic protocols for the HotStuff variants listed in Table 1, our forensic protocol requires only one additional message to identify at least t+1 Byzantine nodes. Furthermore, we demonstrate in the subsequent sections that Abraham et al.’s BB protocol cannot provide forensic support when the number of Byzantine nodes exceeds 2t−2, which indicates that the forensic protocol designed in this paper is optimal in terms of the number of allowable Byzantine nodes.

As a generalization, the contributions of this paper are as follows:

- Designing a forensic protocol for Abraham et al.’s BB. This protocol can label Byzantine nodes that violate the flow of Abraham et al.’s BB when the number of Byzantine nodes controlled by the adversary does not exceed 2t−2, and provide verifiable evidence of specific malicious operations executed by the labeled nodes at each step.
- Analyzing two key performance indicators of the forensic protocol: the number of Byzantine nodes that can be labeled and the number of additional messages that need to be requested. A higher number of labeled Byzantine nodes indicates stronger forensic support, while fewer additional messages indicate greater efficiency. The forensic protocol designed in this paper can label t+1 Byzantine nodes, requiring only one additional message to be transmitted.
- Proposing and proving an impossibility result, demonstrating that Abraham et al.’s BB cannot provide any effective forensic support when the number of Byzantine nodes exceeds 2t−2. This impossibility result shows that the forensic protocol designed in this paper is optimal in terms of the number of allowable Byzantine nodes.

This paper is organized as follows: Section 2 introduced Byzantine broadcast and discusses related work, Section 3 describes the system model, Section 4 reviews Abraham et al.’s BB protocol, and Section 5 presents the forensic protocol for Abraham et al.’s BB and analyzes it. Section 6 provides a proof of the impossibility result supported by Abraham et al.’s BB forensic protocol. Finally, Section 7 concludes the paper.

## 2. Preliminaries and Related Work

This section introduces Byzantine Broadcast, as well as the related work on state machine replication protocols and forensic techniques.

### 2.1. Byzantine Broadcast

Byzantine Broadcasting is used to achieve consistent message delivery in distributed systems, even in the presence of Byzantine nodes, thereby ensuring that all honest nodes reach a consensus on the received message. In such networks, a designated broadcast node is responsible for broadcasting input messages to other nodes. Byzantine broadcasting is specifically defined as follows.

 **Definition 1**  (Byzantine Broadcast (BB) [4])**.** *A Byzantine broadcast protocol is required to fulfill the following properties.*

- *Agreement. It holds that $v=v^{\prime }$, when two honest parties commit value v and $v^{\prime }$, respectively.*
- *Validity. For any honest broadcaster, all honest parties will commit the value sent by the designated broadcaster and terminate.*
- *Termination. Every honest party commits and terminates.*

Since Abraham et al.’s BB employs partially synchronous Byzantine broadcasts, we first provide a brief overview of the adversary capabilities in the partially synchronous network model. Specifically, the adversary can arbitrarily delay messages in the network, with a delay bound of Δ for any message after the Global Stable Time (GST) is reached. A partially synchronized Byzantine broadcast is defined as follows.

 **Definition 2**  (Partially Synchronous Byzantine Broadcast (PSYNC-BB) [4])**.** *A Partially synchronous Byzantine broadcast protocol is required to fulfill the following properties.*

- *Agreement. Same as above.*
- *Validity. For any honest broadcaster, all replicas will commit the value broadcasted by the designated broadcaster, if GST=0.*
- *Termination. After GST is reached, all honest replicas commit and terminate.*

### 2.2. Related Work of State Machine Replication Protocol

Consensus protocols can be categorized into crash fault-tolerant (CFT) and Byzantine fault-tolerant (BFT) protocols based on the type of fault-tolerant node. CFT protocols permit nodes to crash but do not allow them to exhibit malicious behavior, whereas BFT protocols allow nodes to engage in arbitrary malicious actions.

Paxos [12] is one of the most widely used CFT protocols and is deployed in many distributed systems [13,14]. However, the high complexity of the Paxos protocol makes its implementation challenging [15]. The Raft protocol [16], another CFT protocol, simplifies Paxos, reduces implementation difficulty, and is widely used in database systems [17,18].

PBFT [5], proposed by Castro and Liskov, is the first practical Byzantine consensus protocol. In the good case wherein the leaders are honest and the network is synchronized, the PBFT protocol requires only three rounds of interactions to reach consensus. Subsequently, Martin et al. optimized the performance of the Byzantine protocol in this good case by proposing the FaB Paxos protocol, which reduces the number of interaction rounds to two. However, at that time, the protocol had a fault tolerance threshold of n≥3t+1. Martin et al. claimed in their paper that this was the optimal fault tolerance threshold for a two-round Byzantine protocol. This conclusion has been widely accepted for over a decade. However, at the PODC conference in 2021, Abraham et al. systematically analyzed the round complexity of Byzantine protocols in the good case, presenting a two-round Byzantine broadcast protocol, Abraham et al.’s BB, with a fault tolerance threshold of n≥5t−1. Furthermore, Abraham et al. proved that the optimal fault tolerance threshold for two-round Byzantine protocols is n≥5t−1.

In 2023, Giridharan et al. proposed a novel chained BFT protocol, BeeGees [19], which prevents quorum certificate (QC) conflicts by using Prepare messages. This makes it possible to commit operations even when honest leaders are non-consecutive. BeeGees has quadratic word communication complexity when using threshold signatures. When using SNARKs, it achieves linear word communication complexity and responsiveness between consecutive honest leaders. In 2024, Giridharan et al. [20] introduced Autobahn, a high-throughput BFT protocol, to address the trade-off between low latency, fault resilience, and high throughput in partially synchronous networks. Autobahn combines a highly parallel asynchronous data dissemination layer with a low-latency, partially synchronized consensus mechanism. This design enables fast recovery after failures without performance degradation.

### 2.3. Related Work of Forensic Support

In recent decades, researchers have focused on enhancing the security of Byzantine fault-tolerant consensus protocols. However, these protocols do not provide unconditional security and must impose restrictions on the adversary’s capabilities, such as limiting the number of Byzantine nodes under the adversary’s control. To address this issue, researchers have begun developing forensic support for Byzantine fault-tolerant consensus protocols, with the goal of labeling Byzantine nodes that exhibit malicious behavior and providing verifiable evidence when the protocol violates security.

The concept of forensic support in distributed systems was first introduced by Haeberlen et al. [21] in 2007. Tang et al. proposed a Raft-oriented forensic protocol called Raft-Forensics [22]. Although Raft is a consensus protocol that only tolerates crash failures, Tang et al. argued that Byzantine nodes may still appear during its actual operation. Raft-Forensics can identify these Byzantine nodes and achieves performance similar to that of Raft. Civit et al. introduced a Byzantine protocol called Poly-graph [23], where honest nodes can reach consensus as long as the proportion of Byzantine nodes remains below one-third. If the protocol violates agreement, it can label at least one-third of the Byzantine nodes. Sheng et al. [9] analyzed the forensic support of several typical Byzantine protocols, including PBFT [5], HotStuff [24], Algorand [25], and LibraBFT [26], as well as their different versions. They parameterized the forensic support capabilities using three key performance metrics: the number of Byzantine nodes that can be labeled, the number of additional messages required for the forensic protocol, and the maximum number of Byzantine nodes that can be tolerated while still providing forensic support. This paper uses the same metrics to analyze the forensic protocol of Abraham et al.’s BB.

With the development of blockchain systems in recent years, forensic support has become a critical topic in proof-of-stake-based blockchains. Forensic support enables the identification and punishment of nodes exhibiting malicious behavior, typically by reducing their deposits. In the Casper blockchain protocol [26], forensic support is established as one of its security goals, allowing the protocol to label up to one-third of the Byzantine nodes. Although the Casper protocol offers forensic support, it does not guarantee termination. In 2020, Ranchal-Pedrosa and Gramoli proposed a blockchain protocol [27] that can recover from forks by excluding failed nodes. However, this protocol can only ensure the exclusion of crash-failed nodes, not Byzantine-style failures. Additionally, Tsa et al. [28] proposed a forensic protocol for Rollups [29], currently the most widely used form of extended blockchain, which provides verifiable evidence to label faulty nodes.

In recent years, Sheng et al. [30] introduced the concept of transition certificate to address the issue that blockchain protocols cannot simultaneously achieve Player-replaceability and strong forensic support. Based on this concept, they designed a BFT protocol that supports both player-replaceable and maximum forensic support.

## 3. Model Assumptions and Problem Description

This section provides a detailed description of the system model, including assumptions about the adversary’s capabilities, the cryptographic primitives used, and the network environment in which the protocol operates. Additionally, it outlines the problem of forensic support for the Byzantine Broadcasting Protocol and presents the relevant definitions.

Before proceeding with the description, we first define all the symbols, see Table 2.

### 3.1. System Model

The system considered in this paper consists of a total of n=5t−1 nodes, where *t* denotes the maximum number of Byzantine nodes the system can tolerate. Each node has an identifier i∈[n]. The number of nodes actually controlled by the adversary is denoted as *f*. These adversary-controlled nodes are referred to as Byzantine nodes, while the remaining nodes are considered honest. Once an adversary takes control of a node, it gains access to all the internal states of that node and can use it to perform arbitrary malicious operations, such as selectively sending messages to certain nodes or sending different messages to different nodes.

In this paper, we assume the presence of a public key infrastructure (PKI) in the system, where each node has a pair of public and private keys (pk,sk). The membership and public key information of all nodes in the system are known. When a node sends a message *m*, it signs the message with its private key, and the signed message is denoted as ${\langle m\rangle }_{i}$. Upon receiving a message ${\langle m\rangle }_{i}$ from a node, other nodes will use the signature verification algorithm to check the correctness of the signature.

The network model considered in this paper follows the semi-synchronous network model [31]. In this model, it is assumed that there is a known upper bound Δ on message transmission delay and an unknown global stable time (GST). Prior to the GST, the network is asynchronous, with no upper bound on message transmission delay. After the GST, the network becomes synchronous, and the transmission delay between nodes is at most Δ. Additionally, nodes are connected through a peer-to-peer, authenticated, and reliable channel. Messages transmitted over this channel are neither discarded, copied, nor modified. It is important to note that the system model considered in this paper is identical to that of Abraham et al.’s BB protocol.

### 3.2. Forensic Support

A secure Byzantine broadcast protocol provides agreement guarantees when f≤t, meaning that if one honest node outputs the broadcast value *v*, no other honest node will output a different value $v^{\prime }\neq v$. However, the protocol may fail to provide guarantees of agreement when f>t. In this paper, we focus on forensic support, which allows Byzantine nodes to be labeled by the forensic protocol in the event of a agreement violation during the operation of the Byzantine protocol.

To provide forensic support, this paper considers the following execution environment: After executing Abraham et al.’s BB protocol and obtaining the output value, a node returns the output value to the client. If the client detects two conflicting output values, it launches the forensic protocol. The client then sends these conflicting output values to all the nodes and waits for their replies. Some of the nodes execute scripts that may contain the information needed to label Byzantine nodes, and these nodes send their scripts to the client. Upon receiving a reply from a node, the client constructs evidence based on the received script and labels the Byzantine node accordingly.

To formally define the forensic support provided by the Byzantine protocol, this paper adopts the definition of Sheng et al. [9] and uses the ternary (m,k,d) to describe the forensic support. The specific definition is provided below:

**Definition 3**  ((m,k,d)-Forensic support [9]). *Given a Byzantine protocol *Π*, when t<f≤m and two honest nodes output different values, the Byzantine protocol *Π* is said to provide (m,k,d)-forensic support if there exists a forensic protocol that can generate evidence based on the running scripts of k nodes, labeling at least d Byzantine nodes.*

Parameters *m*, *k*, and *d* in the above definition correspond to the constraints of forensic support, forensic efficiency, and forensic capability, respectively. Specifically, parameter *m* defines the maximum number of Byzantine nodes that the forensic support can accommodate. If the actual number of Byzantine nodes in the system exceeds *m*, the protocol may fail to provide forensic support. Parameter *k* reflects the operational efficiency of the forensic protocol: a smaller *k* means fewer messages are required to identify Byzantine nodes, indicating higher efficiency. Parameter *d* indicates the number of Byzantine nodes that the forensic protocol can label. A larger *d* signifies stronger forensic capability and the ability to label more Byzantine nodes.

## 4. Review of Abraham et al.’s BB Protocol

To describe Abraham et al.’s BB forensic protocol in more detail, this section reviews Abraham et al.’s BB protocol. Section 4.1 outlines the operational flow of Abraham et al.’s BB protocol, and Section 4.2 analyzes the agreement guarantees provided by the protocol.

### 4.1. Overview of the Protocol

In Abraham et al.’s BB protocol, a designated broadcast node sends a broadcast value to all other nodes. The remaining nodes output the broadcast value after reaching consensus. It is important to note that all messages in the protocol are authenticated using digital signatures.

The execution of Abraham et al.’s BB protocol is described below. It is divided into views, with each view having a leader node. In view 1, the leader node serves as the broadcast node. In each view, the node performs the following phases:

1. Proposal Phase: The leader of the current view sends a proposal message to all nodes containing the broadcast value *v* and a status certificate *M*. The specific definition of the status certificate is provided later in this section.
2. Voting Phase: Upon receiving a proposal message from the leader, a node verifies that the broadcast value *v* in the proposal message complies with the protocol rules, i.e., whether it aligns with the state certificate *M*. If it passes the verification, the node broadcasts a voting message for *v*.
3. Confirmation Phase: Upon receiving 4t−1 votes for *v* from different nodes in the current view, the node generates a valid Confirmation certificate, output *v*, and returns a reply message to the client. The reply message contains the broadcast value and the corresponding Confirmation certificate.

With a stable network state and an honest leader, the node can successfully complete the above three phases and output the broadcast value. However, if the node fails to output the broadcast value within a reasonable time, it enters the view-switching phase, attempting to change the leader and transition to a new view.

During the execution of Abraham et al.’s BB protocol, leaders from different views may propose conflicting broadcast values. To ensure agreement, the protocol must guarantee that, once an honest node outputs a broadcast value *v* in one view, no remaining node outputs a conflicting value $v^{\prime }$ in later views. Abraham et al.’s BB protocol achieves this agreement through “locking” rules. Specifically, an honest node “locks” a broadcast value, which may be confirmed during a view switch. In subsequent views, a node will only vote on broadcast values that are locked. A node will vote on a different broadcast value only if it receives a status certificate confirming that the most recently locked value of a sufficient number of nodes is not *v*. To implement this locking mechanism, Abraham et al.’s BB protocol introduces timeout certificates, status certificates, and locking rules. The next section provides a brief overview of timeout certificates, status certificates, and the locking rules used in the protocol.

#### 4.1.1. Timeout Certificates and Locked Broadcast Values

When a node enters the view-switching phase, it broadcasts a timeout message containing the broadcast value it has voted for in the current view. If the node has not voted in the current view, the timeout message contains “⊥”. A timeout certificate for the current view is formed when a node receives timeout messages from 4t−1 distinct nodes. By analyzing the timeout messages within the timeout certificate, a node can determine whether to “lock” a broadcast value. Specifically, a timeout certificate locks the broadcast value *v* in one of the following two cases:

1. The timeout certificate contains at least 2t−1 timeout messages that include the broadcast value *v*, and there are no timeout messages that contains a value $v^{\prime }$ that conflicts with *v*.
2. The timeout certificate contains at least 2t timeout messages that include the broadcast value *v*, with no timeout messages from the leader of the current view.

If one of the above two conditions is satisfied, the timeout certificate will lock the broadcast value *v*. If neither condition is satisfied for any value, the timeout certificate will not lock any value. When f≤t, Abraham et al.’s BB protocol guarantees that if a broadcast value *v* is confirmed by a node in view *e*, the timeout certificate for view *e* will necessarily lock the broadcast value *v*.

#### 4.1.2. Status Certificate

After a node generates a timeout certificate for the current view, it sends a status message to the leader of the next view, containing the node’s latest locked value and the corresponding timeout certificate. The leader of the new view collects 4t−1 status messages and sets its broadcast value to the latest locked value from these 4t−1 status messages. Simultaneously, these 4t−1 status messages form a status certificate. The proposal message generated by the new view leader includes its broadcast value and the status certificate. Upon receiving the proposal message, a node can verify that the leader’s broadcast value is the latest locked value from the 4t−1 nodes based on the status certificate.

### 4.2. Agreement Guarantee

The Agreement guarantee analysis of Abraham et al.’s BB protocol involves two aspects: agreement within a single view and agreement across views. These two aspects are described separately below:

#### 4.2.1. Agreement Within a Single View

Within a view, the protocol ensures agreement through voting. Since a node can only vote once, specifically on the first broadcast value it receives from the leader, an honest node can vote only once in each view. If two honest nodes output different broadcast values within the same view, it implies that both broadcast values have corresponding confirmation certificates. Applying the pigeonhole principle, we derive the following formula:

(1) (4t−1)+(4t−1)−n=3t−1,

Equation (1) shows that, to generate confirmation certificates for two conflicting broadcast values within the same view, at least 3t−1 nodes must vote for both values. Thus, when f≤t, the protocol ensures that nodes cannot generate separate confirmation certificates for conflicting broadcast values within the same view, guaranteeing that honest nodes will not output conflicting values within the same view.

#### 4.2.2. Agreement Across Views

Abraham et al.’s BB protocol ensures agreement across views through a locking mechanism. If a node outputs the value *v* in view *e*, then at least 4t−1 nodes must vote on *v* in view *e*. The timeout certificates of all honest nodes in view *e* are locked when f≤t. In this case, all timeout certificates generated by honest nodes in view *e* lock *v*. In subsequent views, honest nodes will not vote for a value $v^{\prime }\neq v$ unless the received state certificate contains a timeout certificate that locks $v^{\prime }$, and the timeout certificate corresponds to a view $e^{\prime }\neq e$. However, generating a timeout certificate to lock $v^{\prime }$ in view $e^{\prime }$ requires that honest nodes vote for $v^{\prime }$ in view $e^{\prime }$. Therefore, Abraham et al.’s BB protocol ensures agreement across views by guaranteeing that when an honest node outputs a broadcast value *v* in view *e*, the remaining nodes will not vote for a conflicting value $v^{\prime }\neq v$ in subsequent views.

## 5. Forensic Protocols for Abraham et al.’s BB

Abraham et al.’s BB protocol provides agreement guarantees only when f≤t. When the number of adversarially controlled nodes exceeds *t*, the protocol may experience agreement violations. This section outlines how to enable Abraham et al.’s BB protocol to provide forensic support in the event of a agreement violation. Specifically, when the number of adversarially controlled nodes f>t and an honest node outputs two conflicting values *v* and $v^{\prime }$, the forensic protocol presented in this paper can provide verifiable evidence that labels the Byzantine node and identifies the step where the Byzantine node deviated from the correct execution of Abraham et al.’s BB protocol.

### 5.1. High Level Overview

This section outlines the high level overview of Abraham et al.’s BB forensic protocol, assuming that the adversary controls f≥t+1 nodes and causes honest nodes to output two conflicting values, *v* and $v^{\prime }$. Depending on whether honest nodes outputs *v* and $v^{\prime }$ in the same view, we consider the following two scenarios:

Case 1: Honest nodes output v and v′ in the same view. In this case, there must exist two confirmation certificates that cause the honest node to output *v* and $v^{\prime }$. From Equation (1), it follows that the intersection of the sets of nodes corresponding to these two confirmation certificates contains at least 3t−1 nodes. These nodes vote for both broadcast values, *v* and $v^{\prime }$, simultaneously in the same view. Therefore, all these 3t−1 nodes violate the protocol flow and can be labeled as Byzantine. The two confirmation certificates for broadcast values *v* and $v^{\prime }$ serve as evidence to label these 3t−1 Byzantine nodes.

**Figure 1:**
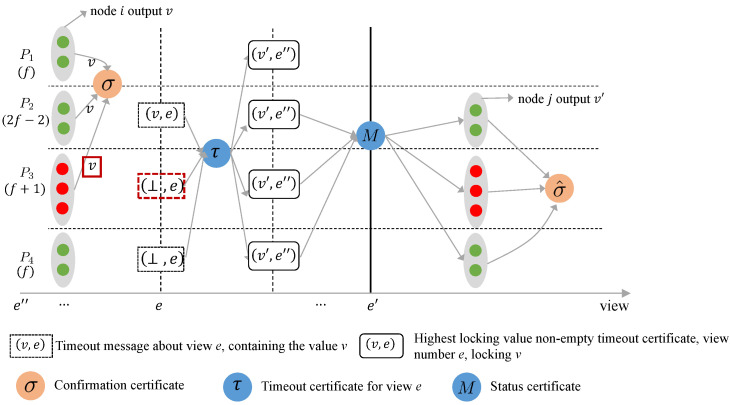
Schematic diagram of honest nodes outputting different values in Abraham et al.’s BB protocol.

Case 2: Honest nodes output v and v′ in two views e and e′, respectively. During the execution of Abraham et al.’s BB protocol, an adversary can cause the protocol to violate agreement by performing a series of operational steps. For clarity, Figure 1 illustrates a potential adversary strategy. The nodes are divided into four sets: $P_{1}$, $P_{2}$, $P_{3}$, and $P_{4}$, where $|P_{1}|\;=\;1$, $|P_{2}|\;=\;2t-2$, $|P_{3}|\;=\;t+1$, and $|P_{4}|\;=\;t$.

1. In view *e*, the nodes in $P_{1}$, $P_{2}$, and $P_{3}$ vote for *v*. A node *i* in $P_{1}$ receives these 4t−1 votes, generates an confirmation certificate, and outputs *v*. Meanwhile, the node in $P_{4}$ does not receive a proposal message from the leader and does not vote for any value in view *e*.
2. At the end of view *e*, all nodes receive timeout messages from $P_{2}$, $P_{3}$, and $P_{4}$. The timeout message for the node in $P_{2}$ contains its vote value *v*, while the timeout messages for the nodes in $P_{3}$ and $P_{4}$ contain ⊥. As a result, all honest nodes fail to meet the protocol’s locking rules for the timeout certificates they generate in view *e*, and thus do not lock any values. The honest node’s most recent timeout certificate with a non-empty locking value has view number $e^{\prime \prime }<e$ and locks $v^{\prime }$.
3. In the subsequent view $e^{\prime }>e$, the 4t−1 timeout certificates received by the leader lock $v^{\prime }$. Therefore, the leader sets its proposal value to $v^{\prime }$ and includes the status certificate consisting of these 4t−1 timeout certificates in the proposal message. According to the protocol’s voting rules, the proposal value set by the leader passes the node check. All nodes then vote on $v^{\prime }$ after receiving the proposal and output $v^{\prime }$.

The reason why the above sequence of events causes the protocol to violate agreement is that the Byzantine node in $P_{3}$ voted for the broadcast value *v* in view *e* and then maliciously included ⊥ in the timeout message in view *e*. This action prevented the honest node from locking any value in the timeout certificate generated in view *e*. The conflicting operations of these Byzantine nodes are highlighted with red boxes in Figure 1.

To forensically verify Abraham et al.’s BB protocol, the client can label at least t+1 Byzantine nodes by collecting the confirmation certificates for the broadcast value *v* in view *e* and the timeout certificates generated by the nodes in view *e*. The client then calculates the intersection of the sets of corresponding nodes from these two certificates. Since the reply message from the honest node already contains the confirmation certificate for the broadcast value *v*, the client only needs to request one additional message: the timeout certificate for view *e* that does not lock any value.

### 5.2. Forensic Protocol

This section provides a detailed description of the forensic protocol proposed in this paper, which enables the labeling of Byzantine nodes in the event of a agreement violation in Abraham et al.’s BB protocol. In the protocol description, we use the notation “∗.ViewNumber” to refer to the view number of the certificate ∗. The superscript of a confirmation certificate σ denotes the confirmation value corresponding to the certificate, while the subscript indicates the view number at the time the confirmation certificate is generated. Additionally, we define the intersection of two certificates *A* and *B* (denoted as A∩B) as the collection of nodes whose signature messages are contained in both *A* and *B*.

The forensic protocol for Abraham et al.’s BB is presented in Algorithm 1. Each node participating in Abraham et al.’s BB protocol stores all received messages as scripts and maintains the set *T* of all timeout certificates it receives (line 2). Note that the set *T* includes the timeout certificates contained in the state certificates received by the node. During protocol execution, if the client detects two conflicting reply values, it first checks whether the two values were output in the same view (line 10).
**Algorithm 1:** Abraham et al.’s BB forensics protocol1:As node *P*2:   T←P Receive all timeout certificates3:   **Upon** Receiving messages from the client 〈REQ-PROOF,e,v〉 **do**4:      **for** TC∈T **do**5:         **if** (TC.ViewNumber ==e)∧(TC not lock v) **then**6:            Send message 〈CERT-TIMEOUT,TC〉 to the client7:As Client8:   **Upon** Received two conflicting reply messages **do**9:      Q:=⌀10:      **if** Two conflicting reply messages have the same view number **then**11:         〈REPLY$,e,v,\sigma_{e}^{v}\rangle \leftarrow$ First reply message12:         〈REPLY$,e,v^{\prime },\sigma_{e}^{v^{\prime }}\rangle \leftarrow$ Second reply message13:         $Q=\sigma_{e}^{v}\cap \sigma_{e}^{v^{\prime }}$14:         Output *Q*15:      **else if** Two conflicting reply messages have different view number **then**16:         〈REPLY$,e,v,\sigma_{e}^{v}\rangle \leftarrow$ Reply messages with lower view number17:         Boardcast 〈REQ-PROOF,e,v〉18:         **Upon** Received Message 〈CERT-TIMEOUT,TC〉, it satisfies(TC.ViewNumber ==e)∧(TC not lock v) **do**19:            **if** The timeout message present in TC contains values conflict with*v* **then**20:               Q=Q∪Leader(e)21:            **for** Node $P_{i}\in \left\{TC\cap \sigma_{e}^{v}\right\}$ **do**22:               **if** The timeout message from $P_{i}$ in TC does not contain *v* **then**23:                  $Q=Q\cup P_{i}$24:         Output *Q*

If both values are output in the same view, the confirmation certificates for both output values can serve as evidence to label the Byzantine nodes (lines 10–14). Specifically, by intersecting the two confirmation certificates, it can identify nodes that voted for two different values within the same view. These nodes are then labeled as Byzantine nodes.

If the client receives two conflicting output values in different views, it broadcasts an interrogation request (line 17) to all nodes, asking for a timeout certificate for view *e* that does not lock the output value *v*, may not lock any value, or locks a value $v^{\prime }\neq v$. Upon receiving the interrogation request from the client, each node searches for a timeout certificate that satisfies this condition within the set *T* (lines 3–6) and sends the corresponding timeout certificate to the client. After receiving the timeout certificate TC that satisfies the condition, the client verifies whether any timeout message contains a conflicting value with *v*. If a conflicting value is found, it indicates that the view is inconsistent with the timeout certificate. In this case, the leader of view *e* has proposed two broadcast values simultaneously, and can be labeled as a Byzantine node (line 20). Next, the client checks the set of nodes where the timeout certificate TC and the confirmation certificate $\sigma_{e}^{v}$ intersect. If the timeout message from any node in this set does not contain *v*, the node is labeled as Byzantine (lines 21–23).

### 5.3. Forensic Support Analysis on Abraham et al.’s BB Protocol

This section systematically analyzes the forensic support for Abraham et al.’s BB protocol. First, the forensic protocol described in Algorithm 1 is examined, and it is demonstrated that, when f≤2t−2, Algorithm 1 can label t+1 Byzantine nodes, requiring only one additional message (see Theorem 3 for details). Theorem 3 shows that Abraham et al.’s BB protocol can provide forensic support with parameters (2t−2,1,t+1).

This section first proves a lemma demonstrating that, when n=5t−1 and f<2t−2, an adversary cannot rely solely on the Byzantine nodes under its control to generate a timeout certificate that locks the broadcast value *v* if no honest node votes for *v*. Subsequently, the section analyzes the case wherein honest nodes output conflicting broadcast values either within the same view or across different views.

**Lemma 1.**  *When n=5t−1 and f≤2t−2, for any view e, no timeout certificate can lock v with respect to view e if no honest node votes for v in view e.*

**Proof of Lemma 1.**  Consider any view *e*. If no honest node votes for *v* in view *e*, at most f≤2t−2 timeout messages from Byzantine nodes will contain *v* in the timeout certificate for view *e*. Since the condition for locking *v* is not satisfied, no timeout certificate for view *e* lock *v*. □

The following section discusses the number of Byzantine nodes that can be labeled by Abraham et al.’s BB forensic protocol and the evidence required when honest nodes output conflicting broadcast values, either in the same view or in different views, respectively.

**Theorem 1.**  *The forensic protocol described in Algorithm 1 can label at least 3t−1 Byzantine nodes without requiring the client to request additional messages when n=5t−1, t<f≤2t−2, and there exist two honest nodes outputting conflicting broadcast values v and $v^{\prime }$ in view e, respectively.*

**Proof of Theorem 1.**  When two honest nodes output broadcast values *v* and $v^{\prime }$, respectively, in view *e*, this implies that confirmation certificates for both broadcast values *v* and $v^{\prime }$ are generated simultaneously in the view. Each confirmation certificate contains 4t−1 votes from different nodes. Since

(4t−1)+(4t−1)−(5t−1)=3t−1,

there must be at least 3t−1 intersecting nodes between the confirmation certificate for *v* and the confirmation certificate for $v^{\prime }$. These 3t−1 nodes can be labeled as Byzantine nodes because they voted for both *v* and $v^{\prime }$ simultaneously in view *e*, violating the protocol’s flow.

After honest nodes confirm a broadcast value *v* (or $v^{\prime }$), its reply message to the client will contain the corresponding confirmation certificate. Therefore, the client can directly label the 3t−1 Byzantine nodes based on the two conflicting reply messages, without incurring additional communication overhead. □

**Theorem 2.**  *When n=5t−1, t<f≤2t−2, and two honest nodes output conflicting broadcast values v and $v^{\prime }$ in views e and $e^{\prime }\neq e$, the forensic protocol described in Algorithm 1 can label at least t+1 Byzantine nodes and only requires the client to request one additional message.*

 **Proof of Theorem 2.**  Without loss of generality, assume that $e<e^{\prime }$. Let the confirmation certificate generated by the honest node in view *e* for the broadcast value *v* be denoted as $\sigma_{e}^{v}$, and the confirmation certificate generated by the honest node in view $e^{\prime }$ for the broadcast value $v^{\prime }$ be denoted as $\sigma_{e^{\prime }}^{v^{\prime }}$. The existence of an honest node outputting the broadcast value $v^{\prime }$ in view $e^{\prime }$ implies that at least 4t−1 nodes vote for the broadcast value $v^{\prime }$ in view $e^{\prime }$. Since the adversary controls at most 2t−2 Byzantine nodes, at least one of these 4t−1 nodes must be honest.

Now, assume that after view *e*, the first view e∗, where $e<e^{*}\leq e^{\prime }$, is the first view in which an honest node votes for the broadcast value $v^{\prime }$. Such a view $e^{*}$ must exist because the presence of honest nodes voting for the broadcast value $v^{\prime }$ in at least view $e^{*}$ satisfies the definition of the view $e^{*}$.

Since there exists an honest node voting on the broadcast value $v^{\prime }$ in view e∗, the highest timeout certificate highTC contained in the state certificate of view $e^{*}$ must lock the broadcast value $v^{\prime }$. Otherwise, the honest node would not have voted on $v^{\prime }$. Additionally, by the definition of view $e^{*}$, no honest node votes on $v^{\prime }$ between views *e* and $e^{*}$. According to Lemma 1, it follows that no timeout certificate locking $v^{\prime }$ exists for views with numbers greater than *e* but less than $e^{*}$. Therefore, the view number of highTC can only be less than or equal to *e*. The following cases are considered based on whether the view number of highTC is less than or equal to *e*.

Case 1: the view number of highTC is less than *e*

Based on the fact that the view number of highTC is less than *e*, it follows that the 4t−1 timeout certificates contained in the state certificate of view $e^{*}$ all have view numbers less than *e*. Therefore, these 4t−1 nodes in view *e* generate timeout certificates without locking any value. Since the number of Byzantine nodes controlled by the adversary is f≤2t−2, there is at least one honest node among these 4t−1 nodes. This honest node sends its timeout certificate $TC_{e}$ generated in view *e* to the client. Based on whether there is a timeout message in $TC_{e}$ containing a conflicting value with *v*, two scenarios are considered:

1. There is no timeout message in $TC_{e}$ that contains a value conflicting with the broadcast value *v*. At most 2t−2 timeout messages in $TC_{e}$ contain the broadcast value *v*; otherwise, $TC_{e}$ would lock the broadcast value *v*. Therefore, there are at least 2t+1 timeout messages in $TC_{e}$ containing ⊥. Since
  (2t+1)+(4t−1)−(5t−1)=t+1,
  there are at least t+1 intersecting nodes between the nodes sending these 2t+1 timeout messages and the corresponding nodes in the confirmation certificate $\sigma_{e}^{v}$. These t+1 nodes can be labeled as Byzantine nodes because they both vote on the broadcast value *v* in view *e* and simultaneously contain ⊥ in the timeout message, violating the protocol flow.
2. If there are timeout messages in $TC_{e}$ containing a value conflicting with the value *v*, at most 2t−1 timeout messages in $TC_{e}$ can contain the broadcast value *v*; otherwise, $TC_{e}$ would lock the broadcast value. Therefore, there are at least 2t timeout messages in $TC_{e}$ that do not contain the broadcast value *v*. Since
  2t+(4t−1)−(5t−1)=t,
  there are at least *t* intersecting nodes between the nodes sending these 2t timeout messages and the corresponding nodes in the confirmation certificate $\sigma_{e}^{v}$. These *t* nodes can be labeled as Byzantine because they both vote for the broadcast value *v* in view *e* and simultaneously fail to include *v* in the timeout message, violating the protocol flow. Furthermore, since there are timeout messages in $TC_{e}$ that contain values conflicting with the broadcast value *v*, this indicates that the leader of view *e* has proposed conflicting broadcast values and can also be labeled as a Byzantine node. Thus, the forensic protocol can identify a total of t+1 Byzantine nodes.

Case 2: the view number of highTC is equal to *e*

According to the definition of highTC, this timeout certificate locks the broadcast value $v^{\prime }$. Two cases are considered below, depending on whether there is a timeout message in highTC that contains a value conflicting with the broadcast value $v^{\prime }$:

1. There is no timeout message in highTC that contains a value conflicting with the broadcast value $v^{\prime }$. In this case, the 4t−1 timeout messages in highTC can only contain the broadcast value $v^{\prime }$ or ⊥, but not the broadcast value *v*. Since
  (4t−1)+(4t−1)−(5t−1)=3t−1,
  there exist at least 3t−1 intersecting nodes between the node that sent these 4t−1 timeout messages and the node corresponding to the confirmation certificate $\sigma_{e}^{v}$. These 3t−1 nodes can be labeled as Byzantine nodes because they both vote for the broadcast value *v* in view *e* and simultaneously do not include the broadcast value *v* in the timeout message, violating the protocol flow.
2. There are timeout messages in highTC that contain values conflicting with the broadcast value $v^{\prime }$. In this case, there are at least 2t timeout messages in highTC that contain the broadcast value $v^{\prime }$. Since
  2t+(4t−1)−(5t−1)=t,
  there exist at least *t* intersecting nodes between the set of nodes sending these 2t timeout messages and the set of nodes corresponding to the confirmation certificate $\sigma_{e}^{v}$. These *t* nodes can be labeled as Byzantine nodes because they both vote for the broadcast value *v* in view *e* and simultaneously include the broadcast value $v^{\prime }$ in the timeout message, violating the protocol flow of Abraham et al.’s BB. In addition, the leader of view *e* can also be labeled as a Byzantine node due to issuing conflicting broadcast values. Therefore, the forensic protocol described in Algorithm 1 can label at least t+1 Byzantine nodes.

In each of the cases discussed above, the client needs to receive only one message from the honest node to label the Byzantine node. Specifically, when the view number of highTC is less than *e*, an honest node generates a timeout certificate $TC_{e}$ for view *e* that does not lock any value. In contrast, when the view number of highTC is equal to *e*, the highTC itself locks a broadcast value $v^{\prime }\neq v$. Thus, in both cases, there exists an honest node that generates a timeout certificate $TC_{e}$ for view *e* that does not lock the broadcast value *v*. The client receives a $\langle \mathrm{CERT}-\mathrm{TIMEOUT},TC_{e}\rangle$ message from the honest node. The timeout certificate $TC_{e}$ and the confirmation certificate for the broadcast value *v* will be used as evidence to label the Byzantine node.

Combining the results from the analysis of Case 1 and Case 2 above, the forensic protocol described in Algorithm 1 can label at least t+1 Byzantine nodes when n=5t−1, t<f≤2t−2, and conflicting broadcast values need to be confirmed. Only one additional message is required to be requested by the client. □

**Theorem 3.**  *The forensic protocol described in Algorithm 1 can label at least t+1 Byzantine nodes when n=5t−1, t<f≤2t−2, and conflicting broadcast values v and $v^{\prime }$ are confirmed in views e and $e^{\prime }$, respectively. Only one additional message is required to be requested by the client.*

**Proof of Theorem 3.**  When $e=e^{\prime }$, according to Theorem 1, the forensic protocol can label at least 3t−1 Byzantine nodes without requiring the client to request an additional message. When $e\neq e^{\prime }$, as stated in Theorem 2, the forensic protocol can label at least t+1 Byzantine nodes, and only one additional message needs to be requested by the client.

In summary, the forensic protocol described in Algorithm 1 can label at least t+1 Byzantine nodes and requires only one additional message from the client. □

## 6. Impossible Conclusion Regarding Forensic Support for Abraham et al.’s BB Protocols

A Byzantine fault-tolerant protocol can ensure security only if the number of Byzantine nodes controlled by the adversary, *f*, is less than the protocol’s fault tolerance threshold, *t*. If the number of Byzantine nodes exceeds this threshold, the protocol may fail to maintain security. Therefore, Byzantine fault-tolerant protocols do not offer unconditional security guarantees and must impose restrictions on the adversary’s capabilities.

Forensic protocols can essentially be classified as a subset of Byzantine fault-tolerant consensus protocols, where the input value is the node’s execution trace and the goal is to reach a consensus on identifying the Byzantine nodes responsible for malicious behavior. As with other Byzantine fault-tolerant consensus protocols, forensic protocols cannot unconditionally achieve the objective of labeling Byzantine nodes. They must also impose restrictions on the capabilities or number of adversaries involved.

In Section 6.2 of this paper, when discussing the forensic support provided by the Byzantine Fault Tolerant Protocol, a parameter *m* is introduced. This parameter indicates the maximum number of Byzantine nodes the protocol can tolerate while still providing forensic support. This section explores the upper bound on *m* in the context of forensic support for Abraham et al.’s BB protocol. Theorem 4 demonstrates that *m* can take values up to 2t−2, and no forensic protocol exists for Abraham et al.’s BB protocol when m≥2t−1.

### 6.1. Overview of the Proof

This section provides an intuitive explanation for why Abraham et al.’s BB protocol cannot offer forensic support when the number of Byzantine nodes is greater than or equal to 2t−1, and outlines the reasoning behind the proof of this impossibility.

Consider the following execution environment: The system consists of a total of n=5t−1 nodes, of which 2t−1 are Byzantine. These nodes are divided into four sets: *O*, *P*, *Q*, and *R*, where |O|=|P|=|Q|=t and |R|=2t−1, with all nodes in *R* being Byzantine.

The total number of nodes in *O*, *P*, and *R* is 4t−1, which are capable of voting on the broadcast value *v*, thereby generating an confirmation certificate for *v* and causing an honest node to output *v*. However, in this execution setting, Abraham et al.’s BB protocol does not guarantee that an honest node will only vote for *v* in subsequent views. This is because 2t−1 Byzantine nodes may include conflicting broadcast values, such as $v^{\prime }$, in their timeout messages for subsequent views. Additionally, other honest nodes may include a null value in their timeout messages due to network fluctuations. According to the locking rule of Abraham et al.’s BB protocol, the timeout messages from these 2t−1 Byzantine nodes can form a timeout certificate locking the broadcast value $v^{\prime }$, with 2t timeout messages containing null values.

In subsequent views, the nodes in sets *O*, *Q*, and *R* receive timeout certificates that lock the broadcast value $v^{\prime }$. Since the view number of the timeout certificate locking $v^{\prime }$ is greater than that of the timeout certificate locking *v*, the nodes in sets *O*, *Q*, and *R* in the subsequent view can vote on the broadcast value $v^{\prime }$ to generate an confirmation certificate for $v^{\prime }$.

During the execution described above, two conflicting values, *v* and $v^{\prime }$, are both output, and the nodes in sets *O* and *R* have voted for these conflicting values. At this point, no forensic protocol can label the nodes in *R* as Byzantine. This is because a client running the forensic protocol can receive run scripts from at most 4t−1 nodes. Since Byzantine nodes cannot send run scripts to the client, the client will stop waiting after receiving scripts from 4t−1 nodes and will label the Byzantine nodes based on the content of these scripts. In the scenario described in this section, the client may only receive run scripts from sets *P*, *Q*, and *R*. Based on these scripts, the client cannot determine whether the nodes in set *O* are voting for $v^{\prime }$ because they received a timeout certificate locking $v^{\prime }$, or if they are voting for conflicting values maliciously. A detailed explanation of why clients cannot distinguish between Byzantine and honest nodes will be provided in the proof in Section 6.2.

### 6.2. Proof of Conclusions

**Theorem 4.**  *When n=5t−1 and f≥2t−1, no forensic protocol for Abraham et al.’s BB can label d>0 Byzantine nodes.*

**Proof of Theorem 4.**  Suppose there are 2t−1 Byzantine nodes in the system, and all the nodes are divided into four sets *O*, *P*, *Q*, and *R*, where |O|=|P|=|Q|=t and R=2t−1. To prove the theorem, assume that there exists a forensic protocol capable of labeling d>0 Byzantine nodes. Two distinct execution environments are constructed below, where the identities of the Byzantine nodes differ in each environment.

**Figure 2:**
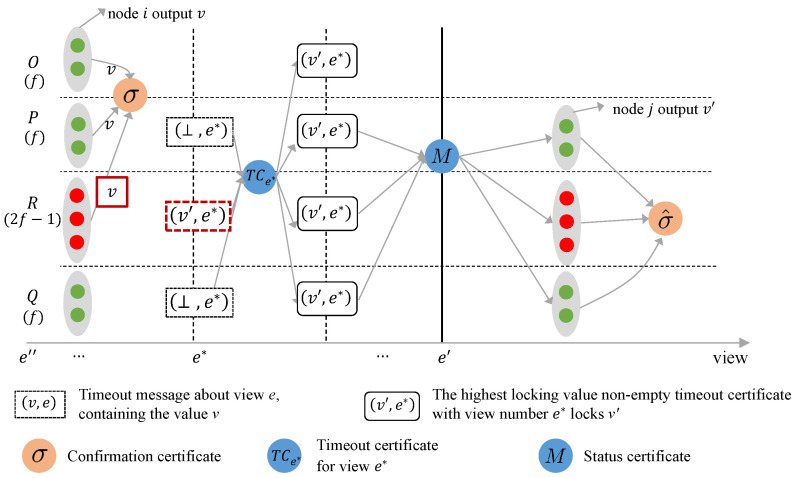
Execution environment I in Theorem 4.

Execution Environment I: Figure 2 presents the schematic diagram of Execution Environment I. In this environment, the nodes in sets *O*, *P*, and *Q* are honest, while the nodes in *R* are Byzantine. Assume that two honest nodes confirm the broadcast values *v* and $v^{\prime }$ in views *e* and $e^{\prime }$, respectively. The confirmation certificates for the broadcast values contain votes from nodes in sets *O*, *P*, and *R*. All nodes generate a timeout certificate that locks the broadcast value *v* in view *e*.

In subsequent views, nodes are unable to receive proposal messages from the leader due to network asynchrony. As a result, all nodes continuously trigger timeouts and broadcast timeout messages containing ⊥. Until view $e<e^{*}<e^{\prime }$, each node receives a timeout message containing $v^{\prime }$ from the 2t−1 Byzantine nodes in *R*, and also receives a timeout message containing ⊥ from nodes in *P* and *Q*. Together, these 4t−1 timeout messages form a timeout certificate $TC_{e^{*}}$ that locks $v^{\prime }$. Subsequently, in view $e^{\prime }$, the highest timeout certificate $TC_{e^{*}}$, contained in the state certificate generated by the leader, is $TC_{e^{*}}$. The nodes in *P*, *Q*, and *R* then vote on the locked value $v^{\prime }$.

During the forensic process, nodes in *O*, *P*, and *Q* send their run scripts to the client. Assuming the existence of a forensic protocol capable of labeling d>0 Byzantine nodes, the nodes in *R* are labeled as Byzantine based on the scripts sent by the nodes in *O*, *P*, and *Q* (which execute the protocol honestly in this execution environment).

**Figure 3:**
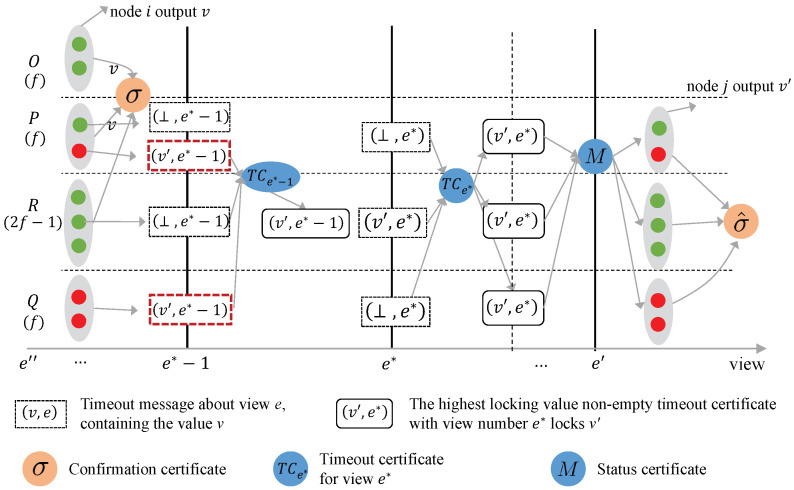
Execution environment II in Theorem 4.

Execution Environment II: Figure 3 illustrates the schematic diagram of execution environment II. In this environment, the sets *O*, *R*, and one node in *P* consist of honest nodes, while the remaining t−1 nodes in *P* and the node in *Q* are Byzantine. It is assumed that two honest nodes confirm the broadcast values *v* and $v^{\prime }$ in views *e* and $e^{\prime }$, respectively. The confirmation certificate for the broadcast value *v* contains votes from nodes in sets *O*, *P*, and *R*. All nodes generate a timeout certificate that locks the broadcast value *v* in view *e*.

In subsequent views, nodes are unable to receive proposal messages from the leader due to network asynchrony, and thus, all nodes continuously trigger timeouts and broadcast timeout messages containing ⊥. By view $e<e^{*}-1<e^{\prime }$, the 2t−1 timeout messages sent by the Byzantine nodes in sets *P* and *Q*, which contain the broadcast value $v^{\prime }$, along with the 2t timeout messages from the honest nodes containing ⊥, form the timeout certificate $TC_{e^{*}-1}$. According to the locking rules of Abraham et al.’s BB protocol, $TC_{e^{*}-1}$ locks the broadcast value $v^{\prime }$. At view e∗, the highest timeout certificate contained in the state certificates received by the nodes in set *R* is $TC_{e^{*}-1}$, prompting the nodes in *R* to vote on the broadcast value $v^{\prime }$ in view e∗ and include $v^{\prime }$ in their timeout messages. During the view changing phase, the timeout messages from the 2t−1 nodes in set *R* contain $v^{\prime }$, while the timeout messages from the Byzantine nodes in sets *P* and *Q* contain ⊥. Together, these 4t−1 timeout messages form a timeout certificate $TC_{e^{*}}$ that locks the broadcast value $v^{\prime }$. In view $e^{\prime }$, the highest timeout certificate contained in the state certificate generated by the leader is $TC_{e^{*}}$. Consequently, the nodes in sets *P*, *Q*, and *R* vote on the locked value $v^{\prime }$ as specified in $TC_{e^{*}}$.

During the forensic process, the Byzantine nodes in sets *P* and *Q* send the same scripts to the client as in execution environment I. The client receives scripts from sets *O*, *P*, and *Q*. As a result, the forensic protocol cannot distinguish between execution environments I and II, and labels the nodes in set *R* as Byzantine nodes. However, in execution environment II, the nodes in *R* are actually honest. Based on the analysis of these two execution environments, it can be deduced that no forensic protocol exists for Abraham et al.’s BB capable of labeling d>0 Byzantine nodes when n=5t−1 and f≥2t−1. □

## 7. Conclusions

This paper investigates the forensic support provided by Abraham et al.’s BB protocol. Abraham et al.’s BB is a Byzantine fault-tolerant broadcasting protocol that achieves two rounds of message exchanges and an optimal fault-tolerance threshold under ideal conditions. However, in comparison to other classic Byzantine fault-tolerant protocols that require three rounds of interactions, Abraham et al.’s BB protocol has a reduced fault-tolerance threshold, from n=3t+1 to n=5t−1. The forensic protocol proposed in this paper is capable of labeling Byzantine nodes with verifiable evidence in the event of a agreement violation in Abraham et al.’s BB protocol. When combined with a punishment mechanism, this forensic protocol can serve as a solution to mitigate the malicious actions of Byzantine nodes. This paper systematically analyzes the proposed forensic protocol and demonstrates that Abraham et al.’s BB protocol can provide (2t−2,1,t+1)-forensic support. Additionally, this paper provides a proof of impossibility, showing that Abraham et al.’s BB protocol cannot offer forensic support when the number of Byzantine nodes controlled by the adversary exceeds 2t−2.

Furthermore, we observe that designing forensic protocols typically requires a comprehensive analysis of the target protocol. Exploring whether one can develop a generalized analytical approach by examining several representative Byzantine broadcast protocols offers an intriguing and valuable direction for future research.

## Figures and Tables

**Table 1:** Comparison of protocol forensic support (*m* represents the number of Byzantine nodes the forensic protocol can tolerate; *k* represents the number of additional messages that need to be sent; and *d* represents the minimum number of Byzantine nodes that can be labeled).

Protocol	*m*	*k*	*d*
HotStuff-view [9]	2t	**1**	t+1
HotStuff-hash [9]	2t	t+1	t+1
Ours	2t−2	**1**	t+1

**Table 2:** Symbol descriptions.

Symbol	Description
*n*	total number of nodes in the system
*t*	maximum number of Byzantine nodes the system can tolerate
*f*	the number of nodes actually controlled by the adversary
(pk,sk)	public and private key for each node
*m* ^1^	message sent by the node
${\langle m\rangle }_{i}$	message signed by the *i*-th node

^1^ In Table 1 and the (m,k,d) triple defined in Definition 3, *m* denotes the number of nodes that the forensic protocol can tolerate, and the protocol description part denotes the information sent by the node.

## Data Availability

No new data were created or analyzed in this study. Data sharing is not applicable to this article.
